# The Effects of Three Modified Whole‐Grain Corn Flours on the Quality of European‐Style Bread

**DOI:** 10.1002/fsn3.71536

**Published:** 2026-02-11

**Authors:** Sun tianying, Wu zhuohao, Ren jian

**Affiliations:** ^1^ College of Food and Biological Engineering Qiqihar University Qiqihar Heilongjiang China

**Keywords:** corn‐based bread, European‐style bread, functional food, modified corn flour

## Abstract

This study investigated the effects of three modification strategies—ultrafine grinding (UGCF), extrusion puffing (ECF), and lactic acid bacteria fermentation (LFCF)—on whole‐grain corn flour and their combined application in European‐style bread. A composite premixed flour system was optimized through single‐factor experiments and an L9 orthogonal design. The optimal formulation consisted of 2.5% ECF, a 5:5 UGCF:LFCF ratio, 7.5% vital wheat gluten, and 45% wheat flour. The resulting corn‐based European‐style bread exhibited a starch content of 66.75% and a dietary fiber content of 9.68%, qualifying it as a high‐fiber product. Antioxidant activity was markedly enhanced, with DPPH and hydroxyl radical scavenging rates reaching 139.65% and 52.35%, respectively. In vitro digestion analysis showed starch hydrolysis degree of approximately 48% after 180 min, accompanied by increased resistant starch content and a calculated glycemic index of 67.45, classifying the product as a medium‐GI food, suggesting an optimal consumption period within 3 days. Overall, the composite modification strategy enabled the development of corn‐based European‐style bread with improved nutritional attributes, enhanced antioxidant activity, and moderated starch digestibility.

## Graphical abstract

The study compared three modification techniques—ultrafine grinding, extrusion puffing, and lactic acid bacteria fermentation—on whole‐grain corn flour for European‐style bread. Modified corn flours improved dough elasticity, cohesion, and bread specific volume while reducing hardness. The resulting corn‐based breads showed higher protein, dietary fiber, and antioxidant activity but lower starch and moisture than wheat‐based bread.

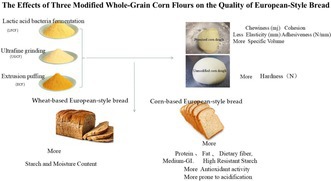

## 1 Introduction

With the continuous improvement of living standards and the growing diversity of food culture, the variety and functionality of bread products have significantly expanded (Cappelli and Cini 2021). In recent years, innovative types such as whole wheat bread, gluten‐free bread, high‐ fiber, or low‐glycemic‐index breads have emerged and gained popularity among health‐conscious consumers (Pico et al. 2017). This trend reflects not only changing dietary preferences. This trend reflects not only changing dietary preferences but also, for certain groups, the necessity of dietary therapy for specific health conditions. For example, individuals with diabetes or those needing better glycemic often require low‐glycemic‐index and high‐fiber breads as part of their dietary management (Szakály and Kiss 2023).

Among the many types of bread, European‐style bread remains a prominent category in the global market (Edima‐Nyah et al. 2024). Characterized by its relatively simple processing techniques, crispy crust, chewy texture, and low sugar, it is regarded as a classic staple with a loyal consumer base. Its adaptability to both artisanal and industrial production has also helped it maintain a strong market presence (Geyzen et al. 2019).

Corn, the most abundantly cultivated grain crop worldwide, is rich in carbohydrates, dietary fiber, essential amino acids, and bioactive compounds such as polyphenols (Xu et al. 2019). However, its application in the baking industry has remained relatively limited, often confined to supplementary roles in multi‐grain or composite flours (Noorfarahzilah et al. 2014). This is mainly due to its weak gluten‐forming ability and differences in starch and protein structure compared to wheat (Hager et al. 2012). At the same time, the distinctive yellow color and strong corn flavor of whole‐grain corn flour may pose sensory challenges, potentially limiting consumer acceptance in bread products. Despite these challenges, whole‐grain corn flour has attracted increasing interest due to its nutritional benefits and gluten‐free properties (Woomer and Adedeji 2021).

To overcome the limitations of conventional whole‐grain corn flour in breadmaking, this study systematically applied three targeted modification techniques—ultrafine grinding (UGCF), extrusion puffing (ECF), and lactic acid bacteria fermentation (LFCF)—to improve the physicochemical and functional properties of corn flour. These techniques were selected because they can be implemented through relatively straightforward processing steps with minimal raw material loss, making them suitable for practical application. Ultrafine grinding primarily reduces corn flour particles to the micrometer scale, significantly increasing the specific surface area and thereby enhancing water absorption and dispersibility, which contributes to improved dough hydration behavior and processing stability. Extrusion puffing induces thermal–mechanical modification under high temperature, high pressure, and intense shear, disrupting starch crystalline structures and promoting partial pregelatinization, which improves water absorption, powder flowability, and mixing uniformity, facilitating rapid dough formation. In contrast, lactic acid bacteria fermentation modifies starch and proteins through microbial metabolism, including organic acid production and enzymatic activity; owing to its more complex biochemical mechanism, fermentation not only improves processing properties but also optimizes nutritional composition and enhances functional attributes.

This study used wheat‐based European bread as a control to assess differences in nutritional composition, antioxidant activity, and starch digestibility, thereby elucidating how these factors affect the quality of corn‐based European bread.

## 2 Materials and Methods

### 2.1 Materials

#### 2.1.1 Preparation of Modified Corn Flour

##### 2.1.1.1 Whole‐Grain Corn Flour (WGCF)

Corn kernels were washed three times with clean water to remove floating and damaged grains, then air‐dried and coarsely ground using a standard grinder (FS‐100 Xulang, China). The resulting flour was sieved through a 200‐mesh sieve to obtain a uniform particle size.

##### 2.1.1.2 Ultrafine Grinding Corn Flour (UGCF)

After washing and air‐drying, the corn kernels were coarsely ground and then subjected to ultrafine grinding for 30 min using an ultrafine milling machine (Zhongkai Powder Machinery Co. Ltd., China). The samples were air‐dried at 45°C in a hot‐air oven until constant weight. The resulting powder was sieved through a 300‐mesh sieve for fine particle distribution.

##### 2.1.1.3 Extruded Corn Flour (ECF)

Corn kernels were washed, air‐dried, coarsely ground, and sieved through a 60‐mesh sieve. The flour was adjusted to a moisture content of 20% and processed using a twin‐screw extrusion‐puffing machine (TS65 Twin‐screw Extruder Saixin Machinery, China) under the following conditions: screw speed of 486 r/min, feed rate of 216 r/min, and barrel temperature of 160°C. The samples were air‐dried at 50°C for 12 h before further analysis. The extruded product was then ground and sieved through a 200‐mesh sieve.

##### 2.1.1.4 Lactobacillus‐Fermented Corn Flour (LFCF)

Cleaned and air‐dried corn kernels were crushed and mixed with deionized water at a solid‐to‐liquid ratio of 1:2. A 0.2% concentration of direct‐vat set lactic acid bacteria was added; *Lactobacillus plantarum* ATCC 8014 was obtained from the China General Microbiological Culture Collection Centre (CGMCC, Beijing, China). The strain was activated in MRS broth at 37°C for 24 h before inoculation. The whole‐grain corn flour was mixed with sterile distilled water at a 1:1 ratio (w/v) and inoculated with activated culture at a final concentration of 10^7^ CFU/mL. The mixture was fermented in a fermentation incubator (Memmert INE 600, Germany) at 37°C for 24 h. After fermentation, the mixture was washed, settled, and sieved through a 200‐mesh sieve.

The bread formulations included modified whole‐grain corn flours (UGCF, ECF, and LFCF), high‐gluten wheat flour and vital wheat gluten (together accounting for 52.5% of total flour weight, with 45% wheat flour and 7.5% vital wheat gluten), eggs (5% w/w), white sugar (2% w/w), edible salt (1.5% w/w), dry yeast (1% w/w), and butter (5% w/w). In functional additives such as bread improver (0.5% w/w), cellulase (135 mg/kg flour), monoglyceride (2% w/w), and sodium carboxymethyl cellulose (CMC, 1.5% w/w) were included to improve dough performance and bread quality. The cellulase (enzyme activity ≥ 10,000 U/g) and other additives, including bread improver, monoglyceride, and sodium carboxymethyl cellulose (CMC), were food‐grade and purchased from Sinopharm Chemical Reagent Co. Ltd. (Shanghai, China). All chemicals and reagents used in analytical measurements (such as ethanol, NaOH, and phenolphthalein) were of analytical grade and supplied by Macklin Biochemical Co. Ltd. (Shanghai, China). Doughs were mixed in a spiral mixer, proofed at 30°C and 75% RH for 60 min, shaped into 250 g loaves, and baked at 180°C for 25 min in a deck oven.

Unmodified corn dough was prepared using unmodified whole‐grain corn flour, combined with wheat flour, vital wheat gluten, and other baking ingredients. This dough was used exclusively for dough‐related analyses and was directly compared with the premixed corn dough prepared from modified corn flours. Comparative evaluations included specific volume determination and compositional measurements (starch, protein, fat, dietary fiber, and moisture content). The unmodified corn dough was not baked into bread and was not subjected to antioxidant or starch digestibility analyses.

Premixed corn dough was prepared using a composite of modified whole‐grain corn flours produced by ultrafine grinding, extrusion puffing, and lactic acid bacteria fermentation, blended with wheat flour and vital wheat gluten. This dough was baked into corn‐based European‐style bread. The resulting bread was compared with wheat‐based European‐style bread, which served as a control, for the evaluation of nutritional composition as well as antioxidant activity and starch digestibility and all analytical measurements were performed in triplicate.

### 2.2 Methods

#### 2.2.1 Particle Size and Powder Flowability Measurements

##### 2.2.1.1 Particle Size Distribution

Particle size distribution was determined using a Microtrac S3500 laser particle size analyzer. Starch samples were suspended in deionized water and ultrasonically dispersed to prepare a 1% (w/w) starch dispersion. A plastic pipette was used to transfer a small aliquot of the dispersion into the instrument's sample cell, and the particle size distribution was automatically analyzed operating in wet measurement mode. The dispersion medium was deionized water, and the corn flour samples were ultrasonically dispersed for 2 min to avoid agglomeration. The instrument, based on Mie scattering theory, has a measurement range of 0.02–2800 μm and an accuracy of 0.6%. The refractive index was set to 1.53, and the obscuration level was maintained between 10% and 15% during measurement.

##### 2.2.1.2 Powder Flowability

The bulk density, tapped density, and angle of repose of the starch were determined using a BT‐1001 intelligent powder characteristics tester. The bulk and tapped densities were calculated according to the following formulas, and the flowability was expressed in terms of the Hausner ratio and Carr's index.

Hausner ratio=Tapped density/Bulk density

Carr'sindex=Tapped density−Bulk density/Tapped density×100%

#### 2.2.2 Nutritional Composition Analysis

The moisture (GB/T 22906.3‐2008, AOAC 925.10), protein (GB/T 5511‐2008, AOAC 979.09), fat (GB/T 5512‐2008, AOAC 922.06), starch (GB/T 5009.7‐2008, AOAC 996.11), and dietary fiber (GB/T 5009.88‐2014, AOAC 985.29) contents of the bread samples were determined following both Chinese national standards and AOAC Official Methods where applicable.

#### 2.2.3 Texture Profile Analysis of Dough

A texture analyzer (TA.XT Plus, Stable Micro Systems Ltd., UK) was used to compare the textural properties of unmodified corn dough and premixed corn dough. Dough samples were prepared into uniform shapes and tested at room temperature.

The test was performed using a double compression cycle with a cylindrical probe (P/36R). The pre‐test speed was 2.0 mm/s, the test speed was 1.0 mm/s, and the post‐test speed was 2.0 mm/s. The compression distance was set to 50% of the sample height. Each sample was tested three times, and the average values were recorded for hardness, cohesiveness, elasticity, adhesiveness, and chewiness.

#### 2.2.4 Specific Volume Measurement

The specific volume of dough made from premixed corn flour and unmodified corn flour was measured using the rapeseed displacement method. After baking and cooling, the loaf volume was determined by the amount of rapeseed displaced, and the weight was measured using an electronic balance. Specific volume was calculated as the ratio of volume (mL) to mass (g). Each sample was tested in triplicate, and the average value was recorded.

#### 2.2.5 Determination of Bread Staling, Acidity, Antioxidant Activity, and Digestibility

Bread staling was evaluated by measuring crumb hardness using a texture analyzer on days 1, 3, 5, and 7 of storage at room temperature. Acidity was assessed according to the Chinese national standard GB/T 5009.17‐2017, using titration with NaOH to determine titratable acidity (°T).

About 10 g of bread crumbs was homogenized in 100 mL of distilled water, filtered, and titrated with 0.1 M NaOH solution using phenolphthalein as an indicator until a faint pink endpoint was reached. The results were expressed as titratable acidity (°T). Antioxidant activity was analyzed using the DPPH and hydroxyl radical scavenging assays. Bread samples were extracted with ethanol, and the scavenging rates were calculated by measuring absorbance at specific wavelengths.

##### 2.2.5.1 DPPH Radical Scavenging Activity

A 2 mL aliquot of the sample extract was mixed with 2 mL of 0.1 mmol/L DPPH solution in absolute ethanol, incubated in the dark for 30 min, and measured at 517 nm ($A_{i}$). The sample blank was prepared by mixing 2 mL extract with 2 mL ethanol (A). The control contained 2 mL DPPH solution + 2 mL ethanol ($A_{0}$). A 0.01 mg/mL ascorbic acid (Vc) solution was used as a positive control. DPPH scavenging activity was calculated as:

$$\text{DPPH scavenging activity}\;\left(\%\right)=\left[1-\frac{A_{i}-A}{A_{0}}\right]\times 100$$

##### 2.2.5.2 Hydroxyl Radical (·OH) Scavenging Activity

A 2 mL aliquot of sample extract (2 mg/mL) was sequentially mixed with 2 mL of 6 mmol/L FeSO_4_ and 2 mL of 6 mmol/LH2O2. After 10 min, 2 mL of 6 mmol/L salicylic acid was added, incubated for 30 min, and measured at 510 nm ($A_{i}$). A parallel reaction without salicylic acid (ethanol substituted for salicylic acid) was recorded as $A_{j}$. The blank control used ethanol instead of the sample and was recorded as $A_{0}$. A 0.01 mg/mL Vc solution was used as a positive control. OH scavenging activity was calculated as:

$$\mathrm{OH}\;\text{scavenging activity}\;\left(\%\right)=\left[1-\frac{A_{i}-A_{j}}{A_{0}}\right]\times 100$$

In vitro starch digestibility was assessed by simulating gastrointestinal digestion. Bread samples were enzymatically hydrolyzed over 180 min, and glucose release was measured at intervals to construct hydrolysis curves. The area under the curve (AUC) was used to estimate the glycemic index (GI).

Starch fractions, including rapidly digestible starch (RDS), slowly digestible starch (SDS), and resistant starch (RS), were determined following the Englyst method, based on glucose release after enzymatic hydrolysis for specific time intervals.

#### 2.2.6 Single‐Factor Experiments of Premixed Flour System

##### 2.2.6.1 UGCF Addition

Added at 2.5%, 5%, 7.5%, 10%, and 12.5% of the total modified corn flour content. UGCF and LFCF Ratio: Blended at 6:4, 5:5, 4:6, 3:7, and 2:8 ratios (excluding ECF) within the modified corn flour content. Vital Wheat Gluten Addition: Incorporated at 7.5%, 10%, 12.5%, 15%, and 17.5% of the total flour weight. WF Addition: Adjusted to 50%, 45%, 40%, 35%, and 30% of the total flour weight.

#### 2.2.7 Wheat Bread Production

A wheat‐based European‐style bread was also prepared using high‐gluten wheat flour without corn flour as a benchmark control. The formulation consisted of 100% high‐gluten wheat flour, eggs (5%), sugar (2%), salt (1.5%), dry yeast (1%), butter (5%), and the same additives used in corn‐based formulations. This control bread was used for comparative analysis of nutritional composition, antioxidant activity, and starch digestibility.

#### 2.2.8 Statistical Analysis

All experiments were conducted in triplicate, and data were expressed as mean ± standard deviation (SD). Statistical analyses were performed using SPSS 26.0 (IBM Corp., USA). One‐way ANOVA followed by Duncan's multiple range test was applied to determine significant differences among groups (*p* < 0.05). For comparisons between two related datasets, a paired sample *t*‐test was used where applicable. Figures were plotted using Origin 2021 and GraphPad Prism 9.

## 3 Results and Analysis

### 3.1 Analysis of Particle Size and Powder Fluidity of Different Modified Corn Flour

**FIGURE 1:**
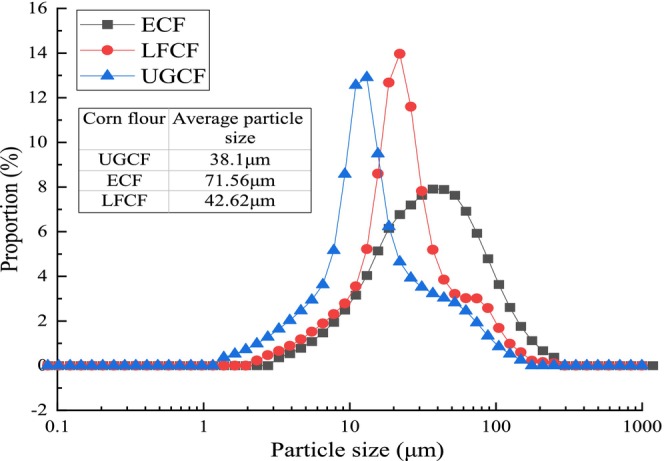
Analysis of particle size distribution of different modified corn flours.

Different modification methods will affect the particle size and powder fluidity of corn flour and then affect the processing characteristics of the dough. The measurement results of particle size and powder fluidity of three kinds of modified whole‐grain corn flour are as follows:

As shown in Figure 1, UGCF (ultrafine‐ground corn flour) exhibited a smaller average particle size of 38.1 μm. In comparison, ECF (extrusion‐puffed corn flour) showed a larger average particle size of 71.56 μm. UGCF and LFCF (lactic acid bacteria‐fermented corn flour) also demonstrated narrower particle size distributions, whereas ECF displayed a broader dispersion. The reduced particle size of UGCF resulted from prolonged ultrafine grinding and subsequent sieving through a 300‐mesh sieve. In contrast, the larger average particle size of ECF was attributed to its wider distribution of coarser particles compared to LFCF, which exhibited a relatively more concentrated particle size profile. Particle size and structural characteristics influence bulk density, tapped density, and angle of repose. The Hausner ratio and Carr's index, derived from the ratio of tapped density to bulk density, serve as indicators of powder flowability. Lower values for these indices signify reduced interparticle cohesion and improved flow properties.

Table 1 indicates that the three modification methods—ultrafine grinding (UGCF), extrusion puffing (ECF), and lactic acid bacteria fermentation (LFCF)—significantly affect the physical properties and flowability of whole‐grain corn flour. UGCF exhibited the highest bulk and tapped densities but showed the poorest flowability, as reflected by its highest angle of repose, Hausner ratio, and Carr's index. In contrast, ECF demonstrated superior flow properties with the lowest angle of repose (38.86°), Hausner ratio (1.12), and Carr's index (11.28%), suggesting that the puffed structure improved powder fluidity. LFCF showed intermediate values, with relatively lower bulk density and moderate flowability. These results indicate that extrusion puffing is most effective in enhancing the powder's flowability, while ultrafine grinding increases particle cohesion, potentially hindering processing performance.

**TABLE 1:** Fluidity analysis of different modified corn flour powders.

	Bulk density	Tapped density	Angle of repose	Hausner	Carr's
UGCF	0.65 ± 0.02a	0.83 ± 0.02a	51.33 ± 1.04c	1.28 ± 0.01a	21.69% ± 0.52b
ECF	0.63 ± 0.03a	0.71 ± 0.03b	38.86 ± 0.86a	1.12 ± 0.01c	11.28% ± 0.48a
LFCF	0.53 ± 0.02b	0.66 ± 0.01c	43.57 ± 0.93b	1.25 ± 0.03b	19.70% ± 1.81b

*Note:* Different superscript letters (a–c) within the same column indicate significant differences (*p* < 0.05).

### 3.2 Effects of Different Modified Corn Flours on Dough Flour Properties

Through the resistance coefficient in the kneading process of dough, the farinaceous characteristics of dough can be characterized and then the processing characteristics of dough can be reflected. The effects of different modified corn flour on farinograph properties are as follows:

Compared with wheat flour (WF), all modified corn flours exhibited significantly higher water absorption, with ECF showing the highest value (79.4 mL/100 g), followed by UGCF (73.8 mL/100 g) and LFCF (71.3 mL/100 g). This indicates that modification treatments increased the water demand of the dough system.

Clear differences were observed in dough development time. UGCF and LFCF significantly prolonged development time to 15.4 and 16.4 min, respectively, compared with 3.6 min for WF, whereas ECF showed a shorter development time (3.0 min). These results demonstrate that ultrafine grinding and fermentation markedly altered the dough mixing behavior, while extrusion puffing did not increase mixing time.

Dough stability time varied substantially among treatments. LFCF exhibited the longest stability time (12.0 min), exceeding that of WF (8.8 min), indicating improved dough resistance to mixing. In contrast, ECF showed a dramatically reduced stability time (1.9 min), reflecting a weak dough structure, while UGCF resulted in a moderate decrease in stability time (7.4 min).

The degree of softening further highlighted structural differences in the dough. ECF exhibited a significantly higher degree of softening (137 EU), more than twice that of WF (57 EU), indicating pronounced weakening during mixing. In contrast, UGCF and LFCF showed lower softening values (52 and 59 EU, respectively), comparable to or lower than WF, suggesting better resistance to mechanical stress.

### 3.3 Single‐Factor Experiments of the Corn‐Based European Bread Premixed Flour System

Single‐factor experiments were conducted to optimize the formulation of a corn‐based European bread premixed flour system composed of three types of modified whole‐grain corn flours, wheat flour, and vital wheat gluten. The study systematically evaluated the effects of varying the proportions of modified corn flours and adjusting the addition levels of wheat flour and vital wheat gluten on dough farinograph properties. Key indicators included water absorption (the optimal water content required for dough formation), development time (the time taken for dough resistance to reach 500 Farinograph Units, FU), stability time (the duration for which the dough maintains a resistance of 500 FU during mixing), and the degree of softening (the reduction in dough resistance after 15 min). These parameters were used to assess the influence of ingredient ratios on dough functionality and processing performance, identify an optimal formulation for producing high‐quality corn‐based European bread.

As shown in Table 3, with the gradual increase in the proportion of ECF in corn flour, the development time, stability time, and degree of softening of the dough all decreased. When the ECF addition reached 5%, the dough stability time dropped to 5.3 min, which still falls within the range typical for medium‐gluten flour. This phenomenon can be attributed to the pregelatinized ECF, which exhibits a loose structure after hydration. The formation of hydrogen bonds between starch molecules creates a gel network, disrupting the cross‐linking between gluten proteins and starch. This weakens the gluten strength, reduces dough stability time, and increases the degree of softening. Additionally, the strong gel‐forming properties of ECF facilitate faster dough aggregation upon water absorption, effectively shortening the kneading process and lowering the development time (Zhu et al. 2023).

As shown in Table 4, increasing the proportion of LFCF (Lactobacillus Fermentation Corn Flour) in corn flour resulted in an initial decrease followed by an increase in dough development time. In contrast, stability time first rose and then declined, accompanied by a consistent upward trend in the degree of softening. The optimal farinograph properties were observed at a 4:6 ratio of UGCF (Ultrafine Grinding Corn Flour) to LFCF, where the dough exhibited the shortest development time (7.6 min) and longest stability time (8.6 min). This improvement stems from LFCF's ability to form strong cross‐links with gluten proteins, enhancing network strength and stability. However, excessive LFCF reduced dough moisture due to its low water‐holding capacity, prolonging development time and accelerating starch retrogradation (via higher amylose content), which hardened the dough and increased softening (Tang et al. 2022). Conversely, UGCF's fine particles effectively filled the protein network, expanding gluten strands and improving stability. Yet, overly high UGCF content caused particle stacking and gluten aggregation, densifying the dough and weakening structural integrity—the 4:6 ratio balanced hydration, gluten‐starch synergy, and particle dispersion, achieving superior dough functionality.

As shown in Table 5, the addition level of vital wheat gluten in the premixed flour significantly influenced the farinograph properties of European‐style bread dough. With increasing vital wheat gluten content, the dough development time exhibited an initial decrease followed by an increase, the stability time first rose and then declined, and the degree of softening initially decreased before rising. At 10% vital wheat gluten addition, the dough achieved the shortest development time, longest stability time, and lowest degree of softening.

Corn flour inherently lacks gluten proteins, making it poorly suited for baked staple products. Vital wheat gluten serves as an exogenous protein supplement, enhancing gluten content in the dough and promoting the formation of a hybrid network between corn starch and gluten proteins, thereby improving gluten strength (Hilhorst et al. 1999). However, excessive gluten addition (> 10%) caused localized aggregation of gluten proteins, disrupting the continuity of the gluten network and reducing stability time. Additionally, over‐added gluten competed for water in the dough, prolonging the kneading process and increasing development time. This highlights the critical balance required in gluten supplementation to optimize dough functionality while avoiding structural compromises.

As shown in Table 6, reducing the proportion of wheat flour (WF) in the premixed flour shortened the dough development time. In contrast, the stability time initially increased before declining, and the degree of softening first decreased, then increased. This is because moderate WF addition complements the gluten protein deficiency in corn flour, fills gaps in the gluten network, and enhances its strength, thereby improving stability and reducing softening (Zhang, Jia, et al. 2023). However, excessive WF forms a coating around corn flour particles, disrupting uniform interactions between corn flour, water, and gluten proteins, which hinders network formation, prolongs development time, and lowers stability. Conversely, insufficient WF leads to inadequate gluten protein content, weakening the network structure and accelerating softening. Optimal WF addition balances gluten reinforcement and particle dispersion for functional dough performance.

### 3.4 Orthogonal Experimental Design

Based on single‐factor experiments, an orthogonal experimental design using an L9 (3^4^) orthogonal array was implemented to optimize the formulation of the corn‐based composite flour system. Table 7 outlines the factor‐level combinations for the orthogonal design, Table 8 presents the experimental results of the orthogonal trials, and Table 9 provides the ANOVA (analysis of variance) results, identifying significant factors influencing dough quality. This systematic approach aimed to determine the optimal ingredient ratios for balancing functional performance and processing efficiency.

As shown in Table 9, using stability time as the evaluation criterion, the order of influence on the range (R) of factors was C > D > A > B, that is, vital wheat gluten > WF > ECF> UGCF: LFCF ratio. The theoretically optimal combination was identified as A1B1C1D1, corresponding to 2.5% ECF, 5:5 UGCF: LFCF ratio, 7.5% vital wheat gluten, and 45% WF. Table 6 revealed that the addition level of vital wheat gluten significantly influenced dough stability time (*p* < 0.05). Verification trials under the theoretical optimal conditions achieved a stability time of 8.5 ± 0.4 min, notably shortening fermentation duration. Thus, the optimal formulation for the corn‐based European bread premixed flour system is 2.5% ECF, 5:5 UGCF: LFCF ratio, 7.5% vital wheat gluten, and 45% WF, ensuring enhanced dough stability and processing efficiency. As shown in Table 9, among the four investigated factors—ECF addition level, UGCF:LFCF ratio, vital wheat gluten content, and wheat flour (WF) content—vital wheat gluten exhibited a highly significant effect on dough stability time, with an F‐ratio of 136.556, which greatly exceeded the critical F‐value of 19.00 (*p* < 0.05). In contrast, the F‐ratios for ECF (9.148), UGCF:LFCF ratio (1.000), and WF (8.889) were all lower than the critical value, indicating that their effects on dough stability time were not statistically significant within the tested factor ranges.

These results demonstrate that, under the conditions of the orthogonal design, vital wheat gluten content was the dominant factor influencing dough stability, whereas the effects of ECF, UGCF:LFCF ratio, and WF were comparatively limited. The relatively small error sum of squares (0.03) further indicates good experimental consistency and reliability of the ANOVA model.

### 3.5 A Comparative Analysis of the Textural Properties Between Corn‐Based Premixed Flour Dough and Unmodified Corn Dough

As shown in Table 10, compared to unmodified corn dough, the premixed flour dough exhibited significantly improved textural properties (except for lower hardness), resembling those of wheat‐based dough. Specifically, the premixed dough demonstrated reduced hardness alongside increased cohesiveness, elasticity, adhesiveness, and chewiness, effectively addressing the shortcomings of unmodified corn dough, such as loose structure, poor viscoelasticity, and undesirable mouthfeel. The unmodified corn dough, retaining the inherent defects of raw corn flour (e.g., low viscoelasticity), displayed weak cohesiveness and adhesiveness, resulting in a crumbly texture. Its excessively low elasticity and chewiness further contributed to a bland, non‐resilient eating quality. In contrast, the premixed system replaced unmodified corn flour with modified variants and incorporated vital wheat gluten, fostering a robust hybrid network between gluten proteins and corn starch. This synergy enhanced structural integrity and optimized textural attributes, resulting in improved dough handling and bread quality comparable to traditional wheat dough, while also providing functional food benefits such as higher dietary fiber content and a lower glycemic response (Zhang, Jia, et al. 2023). Compared with unmodified corn dough, premixed corn dough exhibited significantly improved textural properties (*p* < 0.05), characterized by lower hardness and higher cohesiveness, elasticity, adhesiveness, and chewiness. Unmodified corn dough showed weak cohesiveness and elasticity, reflecting limited viscoelasticity.

### 3.6 A Comparison of the Specific Volume Between Unmodified Corn Dough and Premixed Corn Dough

**FIGURE 2:**
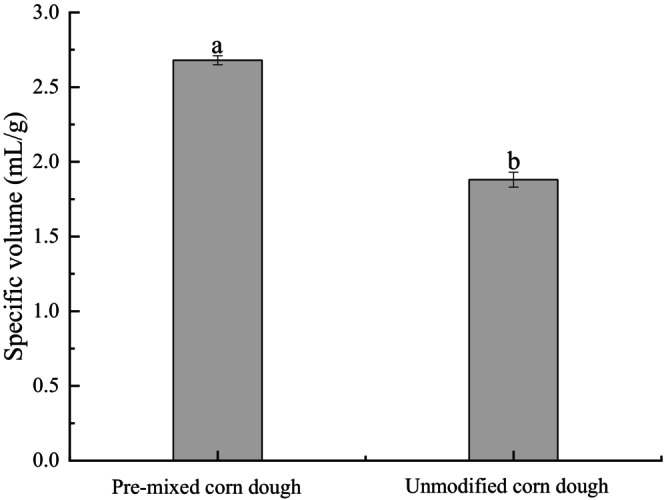
Comparison of the specific volume of premixed corn dough and unmodified corn dough. Letters in the bar graph indicate significant differences among samples (*p* < 0.05).

As shown in Figure 2, significant differences were observed between the premixed corn dough and unmodified corn dough in specific volume (*p* < 0.05). The specific volume of premixed corn dough reached approximately 2.65 mL/g, which was significantly higher than that of unmodified corn dough (about 1.85 mL/g). This result indicates that the composite modification markedly improved the expansion capacity of the dough system.

Specific volume is the ratio of the volume to the quality of the dough. Generally speaking, the larger the specific volume, the larger the volume, the softer the internal structure, and the better the bread taste after proofing. Specific volume pair of premixed flour, corn, and unmodified corn.

As shown in Figure 2, the specific volume of the formulated with the premixed flour system increased significantly by 42.55% compared to the unmodified corn dough. The enhanced specific volume of the premixed system resulted in larger loaf volume, softer texture, and improved consumer acceptance, demonstrating the functional superiority of the optimized formulation.

Specific volume, ratio of bread volume to mass, is a critical quality indicator. Bread with higher specific volume exhibits a stronger gluten network, more uniform gas cell distribution, greater loaf expansion, and softer crumb texture (Monteiro et al. 2021).

### 3.7 Nutritional Composition Analysis of European‐Style Bread

Compared to refined wheat, corn is characterized by lower starch content and higher dietary fiber levels (Svihus 2014). A comparative nutritional dietary analysis was conducted between wheat‐based and corn‐based European bread formulations to verify whether the corn‐based European bread developed in this study retains these nutritional advantages. This evaluation focused on key parameters such as starch, dietary fiber, protein, and fat content, ensuring alignment with corn's inherent nutritional profile while assessing functional enhancements from modified corn flours and gluten supplementation.

Compared to wheat‐based European bread, the corn‐based European bread exhibited a 15.32% reduction in starch content and a 4.5‐fold increase in total dietary fiber. According to the Chinese national standard GB 28050‐2011, a solid food can be labeled as “high in dietary fiber” or “a good source of dietary fiber” if its total dietary content meets or exceeds 6 g/100 g. Experimental results confirm that the corn‐based European bread qualifies as a high‐fiber product. Dietary fiber is recognized for its health benefits, including lowering postprandial blood glucose levels, reducing blood lipids and cholesterol, and improving gut health, making high‐fiber foods valuable functional foods. Additionally, the reduced starch content in corn‐based bread slows the rise of blood sugar levels, aiding glycemic control. Consequently, regular consumption of corn‐based European bread not only enhances satiety but also mitigates rapid glucose spikes, aligning with dietary strategies for metabolic health management (Revilla et al. 2022).

### 3.8 Staling Degree and Acidity Analysis of European‐Style Bread

In Table 12, bread aging during storage was evaluated based on changes in crumb hardness, expressed in Newtons (N), as determined by texture profile analysis. Bread staling is commonly assessed by changes in crumb hardness. When storage exceeded 3 days, the crumb hardness of corn‐based bread exceeded 200 N, leading to a rapid decline in sensory quality and indicating an optimal consumption period within 3 days.

During storage, bread is highly susceptible to staling and acidification, necessitating its consumption shortly after baking. Staling, reflected by increased crumb hardness, and acidity, measured by titratable acid levels, are critical parameters influencing European‐style bread's shelf‐life and storage stability. By analyzing these factors, the optimal storage duration can be determined to balance consumer preferences with practical quality requirements. As shown in Table 12, crumb hardness progressively increased for both corn‐based and wheat‐based European bread during storage. Notably, corn‐based bread exhibited significantly higher hardness and faster hardening due to its higher amylose content, which is prone to retrogradation, causing moisture loss and textural deterioration. After 3 days of storage, the crumb hardness of corn‐based bread exceeded 200 N, accompanied by a marked decline in sensory quality, suggesting that its optimal consumption period should be within 3 days. Similarly, the acidity of both bread types increased over time, with corn‐based bread showing a steeper rise due to lactic acid bacteria introduced during fermentation, which produce additional acidic compounds. Extended fermentation further elevated the acidity. According to the Chinese national standard GB/T 5009.17‐2017, the recommended threshold for bread acidity is ≤ 6°T, a level exceeded by corn‐based bread after 3 days. Therefore, corn‐based European‐style bread is best consumed within 3 days of baking to ensure sensory acceptability and compliance with quality standards.

### 3.9 Antioxidant Activity Analysis of European‐Style Bread

Compared to wheat‐based European bread, the corn‐based European bread demonstrated significantly enhanced antioxidant activity, with DPPH radical scavenging rate and hydroxyl radical scavenging rate increasing by 139.65% and 52.35%, respectively. This improvement is attributed to the inherent functional components of corn, such as polyphenols, flavonoids, and carotenoids, which exhibit potent antioxidant properties. By utilizing whole‐grain corn flour as the primary ingredient, the corn‐based bread effectively retains these bioactive compounds. Regular consumption of such bread may contribute to delaying aging and supporting the management of chronic diseases through its antioxidative effects, aligning with the growing demand for functional foods that offer health benefits beyond basic nutrition (Wichansawakun and Buttar 2019).

### 3.10 Analysis of Hydrolysis Curves in European‐Style Bread

Numerous factors influence the in vitro digestibility of food, with the primary factor being the high temperature during the gelatinization process, which disrupts the crystalline structure of starch, rendering it more susceptible to enzymatic hydrolysis. Additionally, the transient network structures formed by dietary fibers can significantly reduce the accessibility of digestive enzymes, thus starch in high‐fiber foods become more resistant to hydrolysis. This dual mechanism explains why fibers‐rich formulations often exhibit slower starch digestion rates and lower glycemic responses than refined carbohydrate sources (Bohn et al. 2018).

**FIGURE 3:**
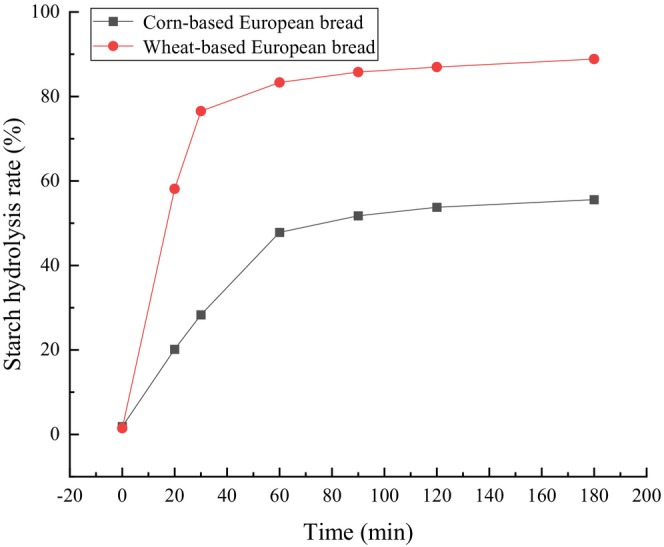
DPPH and hydroxyl radical scavenging activities of corn‐based and wheat‐based European breads. Letters in the bar graph indicate significant differences among samples (*p* < 0.05).

As shown in Figure 3, the starch hydrolysis rates of both corn‐based and wheat‐based European bread increased with simulated in vitro digestion time. Both exhibited rapid increases during the 0–30 min phase, followed by slower rates after 30 min. After 180 min of in vitro starch hydrolysis, the corn‐based European bread reached an estimated starch hydrolysis rate of approximately 48%, which is significantly lower than that of the wheat‐based European bread (82%). This is attributed to the lower starch content, inherently slower digestibility of whole‐grain corn flour, and the higher dietary fiber content in corn‐based bread, which collectively impede enzymatic hydrolysis.

Based on the area under the hydrolysis curve (0–180 min), the glycemic index (GI) of the corn‐based bread was calculated as 67.45. According to the Chinese national standard GB 29922‐2013 classification, foods with a GI between 55 and 75 are categorized as medium‐GI. Thus, the corn‐based European bread qualifies as a medium‐GI food, indicating that its consumption can effectively delay postprandial glucose generation and contribute to long‐term blood glucose management, making it suitable for individuals prioritizing glycemic control (Leahy et al. 2019).

### 3.11 Analysis of Digestive Characteristics in European‐Style Bread

Compared to wheat‐based European bread, the corn‐based European bread exhibited significantly lower rapidly digestible starch (RDS) content and markedly higher resistant starch (RS) content. This is attributed to corn's higher dietary fiber content, which forms a protective fibrous network around starch granules, increasing RS content by nearly 80% relative to wheat‐based bread. Additionally, the higher proportion of amylose and more compact molecular structure in whole‐grain corn flour further contributed to a 118.55% increase in RS content. Consequently, corn‐based European bread demonstrates a slower glycemic response, enhanced satiety, and potential long‐term benefits for lowering blood glucose and cholesterol levels, aligning with dietary strategies for metabolic health management (Revilla et al. 2022).

### 3.12 Corn and Wheat in European Bread

**FIGURE 4:**
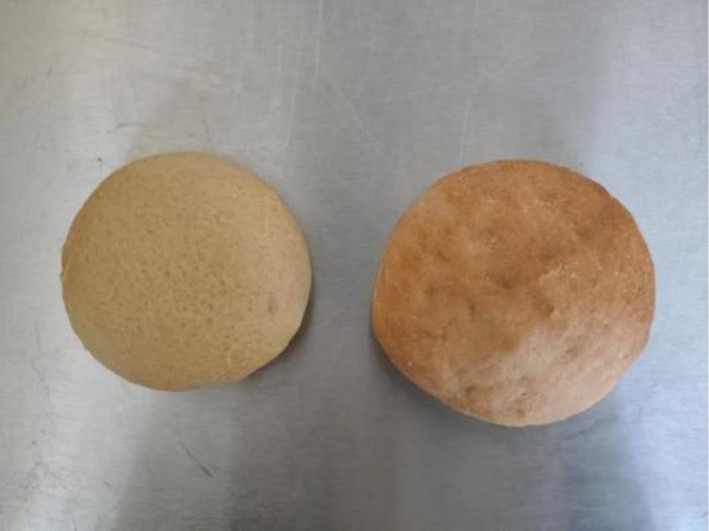
Appearance comparison between wheat‐based (left) and corn‐based European‐style bread (right).

As shown in Figure 4, the corn‐based bread exhibits a visibly larger loaf volume, indicating improved dough expansion. The wheat‐based bread is lighter in color, smoother on the surface, and finer in overall texture.

## 4 Discussion

In Figure 1, the average particle size of UGCF was significantly smaller than that of ECF (38.1 vs. 71.56 μm). In addition, UGCF and LFCF exhibited relatively narrow particle size distributions, whereas ECF showed a much broader and more dispersed distribution. Particle size distribution plays a crucial role in determining the packing behavior and flowability of powders. Owing to prolonged ultrafine grinding and subsequent collection through a 300‐mesh sieve, UGCF particles were markedly reduced in size and more uniformly distributed. The increased number of interparticle contact points and the enlarged specific surface area enhance van der Waals interactions and mechanical interlocking between particles. Such structural characteristics generally increase powder cohesiveness, thereby hindering free flow (Shah et al. 2023).

In contrast, although LFCF and ECF exhibited larger average particle sizes, pronounced differences were observed in their particle size distributions. ECF displayed a broader distribution, particularly with a higher proportion of coarse particles, which further increased its average particle size. A wide particle size distribution can facilitate the filling of voids between larger particles by finer ones, thereby modifying the packing structure and, to some extent, reducing heterogeneity in effective contact areas. However, when the proportion of coarse particles becomes excessive, enhanced geometric interlocking between particles may occur, leading to an unstable packing structure and consequently impairing overall flowability (Ambrose et al. 2016).

In Table 1, bulk density, tapped density, and angle of repose are commonly used macroscopic indicators for characterizing powder flowability, and their variations are closely associated with particle size and structural characteristics. The Hausner ratio and Carr's index reflect the degree of powder consolidation under external forces; generally, lower values indicate weaker interparticle adhesion and better flowability (Chinwan and Castell㏄erez 2019). Therefore, when combined with particle size distribution characteristics, it can be inferred that UGCF, with its smaller particle size and narrower distribution, is more prone to higher cohesiveness and relatively poorer flowability. In contrast, ECF, characterized by larger particle size and a more dispersed distribution, exhibits distinct packing and flow behaviors.

In Table 2 compared with WF, doughs prepared from the three modified flours exhibited significant differences in farinograph characteristics, indicating that different modification strategies exert a decisive influence on dough development and structural stability. The development times of UGCF and LFCF doughs were significantly prolonged, increasing by 11.8 and 12.8 min, respectively, which is closely associated with their enhanced water absorption capacity and water redistribution behavior. During the initial mixing stage, UGCF and LFCF readily absorb water and rapidly agglomerate; however, during subsequent kneading, water must be redistributed under mechanical forces to facilitate the reformation of the internal network structure, thereby delaying the attainment of dough stability.

**TABLE 2:** Effect of different modified corn flours on dough flour properties.

	Water absorption (ml/100 g)	Development time (min)	Stability time (min)	Degree of softening (EU)
WF	64.2 ± 1.0c	3.6 ± 0.2c	8.8 ± 0.8b	57 ± 0.9c
UGCF	73.8 ± 0.9b	15.4 ± 0.8b	7.4 ± 0.7c	52 ± 0.5c
ECF	79.4 ± 1.0a	3 ± 0.2d	1.9 ± 0.1d	137 ± 1.0a
LFCF	71.3 ± 0.9bc	16.4 ± 0.6a	12 ± 1.0a	59 ± 0.8bc

*Note:* Different lowercase letters in the same column indicate significant differences between samples (*p* < 0.05).

In contrast, owing to extrusion‐puffing treatment, ECF exhibited a relatively loosened starch structure, enabling more rapid mixing with gluten and the formation of a starch gel–dominated matrix during mixing. Consequently, the development time of ECF dough was shorter than that of WF. This observation suggests that dough development time is governed not only by water absorption kinetics but also by the nature of starch–protein interactions and the pathway of structural reorganization (Masbernat et al. 2021).

More pronounced differences were observed in dough stability time and degree of softening among the modified flours. The stability time of LFCF dough was significantly extended, indicating enhanced resistance to mechanical shear. This behavior can be attributed to the exposure of abundant hydrophilic groups during lactic acid fermentation, which interact with sulfhydryl and disulfide bonds in gluten upon mixing, thereby reinforcing the continuous gluten network and improving dough stability. Although the stability time of UGCF dough decreased slightly compared with WF, it remained significantly higher than that of ECF. This effect is primarily due to the smaller particle size of UGCF, which increases interparticle adhesion and limits the uniform dispersion of gluten within the system, preventing it from fully filling and supporting the network structure, thus exerting a negative influence on structural stability (Pruett et al. 2023).

ECF dough exhibited the shortest stability time and a markedly increased degree of softening, with the softening degree reaching 2.4 times that of WF. This phenomenon is closely related to starch pregelatinization induced by extrusion‐puffing treatment. Pregelatinization disrupts the native starch granule structure and markedly enhances water absorption; however, hydrated starch tends to form hydrogen bonds predominantly within starch molecules, thereby weakening effective cross‐linking with gluten. In addition, the reduction in short‐range ordered structures in ECF further compromises the structural integrity and shear resistance of the dough system, ultimately resulting in reduced stability time and increased softening.

In Table 3 with the gradual increase in the proportion of ECF in corn flour, the dough development time, stability time, and degree of softening all exhibited a continuous decreasing trend, indicating that ECF content is a key factor influencing dough farinograph behavior. When the ECF addition level reached 5%, the dough stability time decreased to 5.3 min, which still falls within the stability range of medium‐strength flour, suggesting that the adverse effect of ECF on dough processability remains acceptable at low inclusion levels.

**TABLE 3:** Effect of ECF addition in modified corn flour on the dough quality of European bread.

ECF proportion in modified corn flour (%)	2.5%	5%	7.5%	10%	12.5%
Water absorption (%)	77.3 ± 0.9ᶜ	78.4 ± 1.1ᵇ	78.7 ± 1.0ᵇ	79.6 ± 0.8ᵃ	80.7 ± 0.9ᵃ
Development time (min)	8.8 ± 0.4ᵃ	7.8 ± 0.5ᵇ	5.6 ± 0.3ᶜ	5.5 ± 0.2ᶜ	5.4 ± 0.2ᶜ
Stability time (min)	6.9 ± 0.3ᵃ	5.3 ± 0.2ᵇ	4.1 ± 0.1ᶜ	4.0 ± 0.1ᶜ	3.8 ± 0.1ᶜ
Degree of softening (BU)	55 ± 1.0ᵈ	132 ± 2.1ᶜ	150 ± 1.8ᵇ	192 ± 2.3ᵃ	208 ± 2.0ᵃ

*Note:* Different lowercase letters in the same column indicate significant differences among samples (*p* < 0.05).

This trend is closely associated with the pregelatinized starch structure formed in ECF as a result of extrusion‐puffing treatment. Pregelatinization disrupts the native starch granule structure, facilitating molecular chain unfolding upon hydration and promoting the preferential formation of hydrogen bonds among starch molecules, thereby generating a starch‐dominated gel network. Such a gel structure partially hinders effective cross‐linking between gluten proteins and starch (Van Hung et al. 2007), weakens the formation and reinforcement of a continuous gluten network, and consequently reduces gluten strength, as reflected by shortened stability time and a lower degree of softening.

Meanwhile, the strong gelation properties of ECF also exert a pronounced influence on dough development. With increasing ECF content, corn flour tends to agglomerate more rapidly upon water addition, reducing the time required for water redistribution and structural reorganization during mixing and kneading, thereby effectively shortening the dough development time. These results indicate that the incorporation of ECF, to some extent, shifts the dough structure formation pathway from a protein‐dominated network toward a starch gel‐assisted system.

In Table 4 with the gradual increase in the proportion of LFCF in corn flour, the farinograph properties of the dough exhibited a pronounced nonlinear pattern: the dough development time initially decreased and then increased, the stability time first increased and subsequently decreased, while the degree of softening showed an overall increasing trend. These results indicate the existence of an optimal synergy range between UGCF and LFCF, beyond which an imbalance in their proportions adversely affects dough structural stability. When the addition ratio of UGCF to LFCF was 4:6, the dough exhibited the shortest development time and the longest stability time, suggesting that, under this ratio, the efficiency of internal structural reorganization and the stability of the network structure reached a relatively optimal state.

**TABLE 4:** The effect of the ratio of UGCF to LFCF in corn flour on the dough quality of European bread.

Ratio of UGCF to LFCF in corn flour	6∶4	5∶5	4∶6	3∶7	2∶8
Water absorption (%)	78.9 ± 0.8ᵃ	78.3 ± 0.7ᵃᵇ	78.2 ± 0.9ᵇ	77.8 ± 0.6ᵇ	77.4 ± 0.8ᵇ
Development time (min)	9.0 ± 0.5ᵃ	8.0 ± 0.6ᵇ	7.6 ± 0.4ᵇ	7.8 ± 0.5ᵇ	7.9 ± 0.4ᵇ
Stability time (min)	7.6 ± 0.4ᵇ	8.3 ± 0.3ᵃᵇ	8.6 ± 0.4ᵃ	8.3 ± 0.5ᵃᵇ	8.3 ± 0.3ᵃᵇ
Degree of softening (BU)	88 ± 1.3ᵇ	80 ± 1.1ᶜ	106 ± 2.0ᵃ	113 ± 2.4ᵃ	144 ± 3.1ᵃ

*Note:* Different lowercase letters in the same column indicate significant differences among samples (*p* < 0.05).

**TABLE 5:** Effect of gluten content in premix flour on dough quality of European bread.

Proportion of vital wheat gluten in premixed flour (%)	7.5%	10%	12.5%	15%	17.5%
Water absorption (%)	78.4 ± 0.9ᵇ	79.1 ± 0.7ᵃ	76.5 ± 0.8ᶜ	71.5 ± 0.6ᵈ	69.8 ± 0.9ᵈ
Development time (min)	9.8 ± 0.4ᵃ	7.8 ± 0.5ᶜ	8.8 ± 0.3ᵇ	9.6 ± 0.4ᵃ	10.0 ± 0.5ᵃ
Stability time (min)	8.3 ± 0.3ᵃᵇ	8.6 ± 0.2ᵃ	7.9 ± 0.3ᵇ	7.9 ± 0.4ᵇ	7.6 ± 0.3ᵇ
Degree of softening (BU)	76 ± 1.8ᵇ	65 ± 1.2ᶜ	67 ± 1.5ᵇᶜ	69 ± 1.7ᵇ	79 ± 2.1ᵃ

*Note:* Different lowercase letters in the same column indicate significant differences among samples (*p* < 0.05).

**TABLE 6:** Effect of the addition of wheat flour and gluten in premix flour on the dough quality of European bread.

Proportion of WF and vital wheat gluten in premixed flour (%)	50%	45%	40%	35%	30%
Water absorption (%)	78.4 ± 0.9ᵇ	80.1 ± 0.8ᵃ	80.9 ± 0.7ᵃ	80.0 ± 0.6ᵃ	79.1 ± 0.9ᵇ
Development time (min)	9.8 ± 0.4ᵃ	9.8 ± 0.5ᵃ	9.0 ± 0.3ᵇ	8.7 ± 0.3ᵇ	8.6 ± 0.4ᵇ
Stability time (min)	8.2 ± 0.3ᵃᵇ	8.3 ± 0.4ᵃ	8.5 ± 0.4ᵃ	7.8 ± 0.3ᵇ	6.6 ± 0.2ᶜ
Degree of softening (BU)	132 ± 2.1ᵇ	127 ± 1.9ᵇ	135 ± 2.0ᵇ	169 ± 2.6ᵃ	214 ± 3.3ᵃ

*Note:* Different lowercase letters in the same column indicate significant differences among samples (*p* < 0.05).

**TABLE 7:** Orthogonal L9 (34) factor‐level table.

Level	Factor			
	A (ECF)/%	B (UGCF∶LFCF)	C (vital wheat gluten)/%	D (WF)/%
1	2.5	5∶5	7.5	45
2	5	4∶6	10	40
3	7.5	3∶7	12.5	35

**TABLE 8:** Orthogonal experiment results.

Serial number	Factor				Stabilization time (%)
	A	B	C	D	
1	1	1	1	1	8.80
2	1	2	2	2	7.90
3	1	3	3	3	6.90
4	2	1	2	3	7.20
5	2	2	3	1	7.00
6	2	3	1	2	8.30
7	3	1	3	2	7.00
8	3	2	1	3	8.50
9	3	3	2	1	8.00
Mean value 1	7.867	7.667	8.533	7.933	
Mean value 2	7.500	7.800	7.700	7.733	
Mean value 3	7.833	7.733	6.967	7.533	
Range	0.367	0.133	1.566	0.400	

**TABLE 9:** Variance analysis table.

Factor	Sum of squares	Degrees of freedom	F‐ratio	Critical F‐value	Significance
ECF	0.247	2	9.148	19.00	
UGCF∶LFCF	0.027	2	1.000	19.00	
Vital wheat gluten	3.687	2	136.556	19.00	*
WF	0.240	2	8.889	19.00	
Error	0.03	2			

*Note:* *indicates the vital wheat gluten has a significant impact on the dough’s stability time.

**TABLE 10:** Comparison of textural properties between unmodified corn dough and premixed corn dough.

	Hardness (N)	Cohesion	Elasticity (mm)	Adhesiveness (N/mm)	Chewiness (mj)
Unmodified corn dough	2.78 ± 0.08ᵃ	0.10 ± 0.01ᵇ	0.54 ± 0.04ᵇ	0.38 ± 0.02ᵇ	0.20 ± 0.03ᵇ
Premixed corn dough	2.16 ± 0.11ᵇ	0.3 ± 0.05a	1.22 ± 0.35ᵃ	0.63 ± 0.14a	0.8 ± 0.40a

*Note:* Different lowercase letters in the same column indicate significant differences among samples (*p* < 0.05).

**TABLE 11:** Comparison of nutritional components between corn and wheat in European bread (%).

	Starch	Protein	Fat	Dietary fiber	Moisture content
Corn‐based European‐style bread	66.75 ± 2.45ᵇ	15.03 ± 0.86a	5.6 ± 0.48a	9.68 ± 0.86ᵃ	34.94 ± 1.04ᵇ
Wheat‐based European‐style bread	78.83 ± 1.83a	13.86 ± 0.88ᵇ	5.54 ± 0.37ᵃ	1.77 ± 0.64ᵇ	37.71 ± 1.21a

*Note:* Different lowercase letters in the same column indicate significant differences among samples (*p* < 0.05).

**TABLE 12:** Comparison of the aging degree different days between corn and wheat in European bread.

	1d	3d	5d	7d
Wheat based European‐style bread	120.79 ± 12.08ᵇ N	157.94 ± 21.01ᵇ N	226.88 ± 25.04ᵇ N	284.89 ± 32.02ᵇ N
Corn based European‐style bread	184.39 ± 15.11a N	218.16 ± 18.05a N	289.33 ± 26.35a N	345.75 ± 27.14a N

*Note:* Different lowercase letters in the same column indicate significant differences among samples (*p* < 0.05).

At moderate addition levels, LFCF can interact strongly with gluten proteins, promoting the formation of cross‐linking points within the protein network and thereby enhancing the continuity and strength of the gluten matrix, which contributes to an extended dough stability time. However, LFCF exhibits a relatively low water‐holding capacity, and excessive incorporation significantly disrupts the effective water distribution within the dough system, making adequate hydration and dough formation more difficult. As the LFCF content further increases, insufficient available water restricts the extension and reorganization of gluten proteins, leading to a progressive increase in dough development time.

In addition, the relatively high amylose content in LFCF is an important factor affecting dough tolerance to mixing. Amylose is more prone to retrogradation, which promotes moisture migration and loss during mixing and resting, causing the dough to gradually lose elasticity and become firmer. As a result, dough structure degradation is accelerated, manifested as reduced mixing tolerance and an increased degree of softening (Yamauchi et al. 2014).

The incorporation of UGCF can partially mitigate these adverse effects. Owing to its smaller particle size, an appropriate amount of UGCF particles can effectively fill the voids within the gluten network, allowing protein fibrils to fully extend and form a denser and more continuous structure, thereby enhancing the overall stability of the gluten network, prolonging stability time, and reducing the degree of softening (Mccann et al. 2016). However, when UGCF is present at excessive levels, the fine particles tend to stack and aggregate within the dough matrix, leading to localized overconcentration of gluten proteins and an overly compact internal structure, which ultimately hinders proper gluten network development.

In Table 6, the level of gluten addition in the premixed flour exerted a significant influence on the farinograph properties of European‐style bread dough, exhibiting a typical pattern of initial improvement followed by deterioration. With increasing gluten content, the dough development time, stability time, and degree of softening showed pronounced nonlinear responses. At a gluten addition level of 10%, the dough exhibited the shortest development time, the longest stability time, and the lowest degree of softening, indicating that dough structure formation efficiency and structural stability reached a relatively optimal state.

Corn flour inherently lacks gluten proteins, which represents a key limitation for its application in baked staple foods. As an exogenous source of gluten proteins, appropriate supplementation with gluten significantly increases the effective gluten content in the dough system and promotes interactions both among gluten proteins and between gluten proteins and corn starch. These interactions facilitate the formation of a more continuous and stable network structure, thereby enhancing dough strength and improving resistance to mechanical shear during mixing and kneading, which in turn prolongs stability time and reduces the degree of softening.

However, when the level of gluten addition is further increased, its positive effect on dough structure gradually diminishes and may even become detrimental. Excessive gluten tends to form localized aggregates within the dough matrix, hindering the uniform dispersion and effective cross‐linking of gluten proteins and restricting normal gluten network development. As a result, structural integrity is compromised and dough stability time decreases. In addition, gluten exhibits a strong water‐binding capacity and, at high inclusion levels, competes with other components for available water in the dough system, disrupting water distribution and reorganization, increasing mixing resistance, and ultimately prolonging dough development time (Skrabanja et al. 2001).

The level of WF addition in the premixed flour exerted a significant influence on the farinograph properties of European‐style bread dough, following a typical pattern in which an optimal level was beneficial, whereas excessive or insufficient addition was detrimental. As the WF content decreased, the dough development time gradually shortened, while the stability time and degree of softening exhibited trends of initial improvement followed by deterioration. These results indicate that, in corn‐based dough systems, WF not only serves as a source of gluten proteins but that its proportion also directly affects the efficiency of gluten network formation and structural integrity.

At appropriate addition levels, WF can be well integrated with corn flour, supplementing the gluten proteins lacking in corn flour and effectively filling voids within the gluten network. This enhances network continuity and strength, resulting in improved resistance to mechanical shear during mixing, prolonged stability time, and a reduced degree of softening (Zhang et al. 2023). However, excessive WF addition tends to induce an encapsulation effect on corn flour particles, leading to heterogeneous contact among corn flour, water, and gluten proteins. This hinders uniform gluten dispersion and effective cross‐linking, thereby impairing normal gluten network development and manifesting as prolonged development time and reduced stability time. Conversely, insufficient WF addition results in a low overall gluten content, making it difficult to establish a continuous and robust gluten network, which in turn reduces dough stability and increases the degree of softening.

In Table 10, in terms of textural properties, compared with unmodified corn dough, the premixed flour dough exhibited significantly reduced hardness, while cohesiveness, springiness, adhesiveness, and chewiness were all markedly enhanced, yielding a texture more comparable to that of wheat dough. Unmodified corn dough typically suffers from loose structure and poor viscoelasticity due to the inherently low elasticity of corn flour and the absence of an effective protein network, resulting in low cohesiveness, adhesiveness, elasticity, and chewiness and consequently inferior sensory quality. In the premixed flour system, partial or complete replacement of unmodified corn flour with modified corn flour effectively alleviated these structural deficiencies. In addition, the incorporation of gluten promoted the formation of a relatively stable gluten network between gluten proteins and corn starch, thereby substantially improving overall dough textural properties (Chen et al. 2018).

In Figure 2, these improvements in dough structure and rheological behavior were ultimately reflected in the quality of the final European‐style bread. Compared with unmodified corn bread, the specific volume of bread prepared from the premixed flour system increased significantly, by 42.55%. A higher specific volume corresponds to a more developed crumb structure, larger loaf volume, and softer texture, which are conducive to enhanced eating quality and greater consumer acceptance. Overall, rational adjustment of WF addition, in synergy with modified corn flour and gluten supplementation, can markedly improve the processing performance, structural properties, and sensory quality of corn‐based European‐style bread.

Specific volume, defined as the ratio of bread volume to mass, is a key indicator for evaluating bread quality and internal structural status. In general, breads with a higher specific volume exhibit a stronger gluten network, a more abundant and uniform cell structure, a larger loaf volume, and a softer mouthfeel. In this study, the specific volume of corn European‐style bread prepared using the premixed flour system was significantly increased, indicating a marked improvement in internal structure. This enhancement can be attributed to the appropriate addition of gluten and wheat flour, which ensured a sufficient level of gluten proteins in the dough system. Meanwhile, the modified corn flour was able to interact effectively with gluten proteins to form a relatively stable gluten network, thereby efficiently entrapping and retaining carbon dioxide generated during fermentation and baking, ultimately promoting loaf expansion and significantly increasing specific volume.

In Table 11, in terms of nutritional composition, compared with conventional wheat European‐style bread, the corn European‐style bread exhibited a 15.32% reduction in starch content, while total dietary fiber content increased by approximately 4.5‐fold. According to the Chinese national standard GB 28050‐2011, solid foods containing ≥ 6 g/100 g of dietary fiber may be labeled as “high in dietary fiber” or a “good source of dietary fiber.” Based on this criterion, the corn European‐style bread developed in this study can be classified as a high‐fiber bread. Dietary fiber is known to reduce postprandial glycemic response, improve blood lipid and cholesterol profiles, and promote intestinal health (Xiaoman 2001); therefore, high‐fiber foods are widely regarded as functional foods with potential health benefits. In addition, the reduced starch content may effectively slow glucose release in the body, contributing positively to glycemic control. Overall, long‐term consumption of corn European‐style bread may not only enhance satiety but also assist in moderating blood glucose levels.

In Table 12, food staling is commonly characterized by changes in hardness during storage. The results showed that crumb hardness of both corn and wheat European‐style breads increased with storage time; however, the hardness of corn European‐style bread was consistently and significantly higher than that of wheat bread, and its rate of increase was more pronounced. This difference is mainly related to starch composition. Compared with wheat flour, corn flour contains a higher proportion of amylose, which is more prone to retrogradation. As storage time extends, moisture migration intensifies, leading to progressive crumb dehydration and hardening. When storage exceeded 3 days, the crumb hardness of corn European‐style bread surpassed 200 N, at which point sensory quality declined sharply. Therefore, the optimal consumption period for corn European‐style bread should be limited to within 3 days.

**FIGURE 5:**
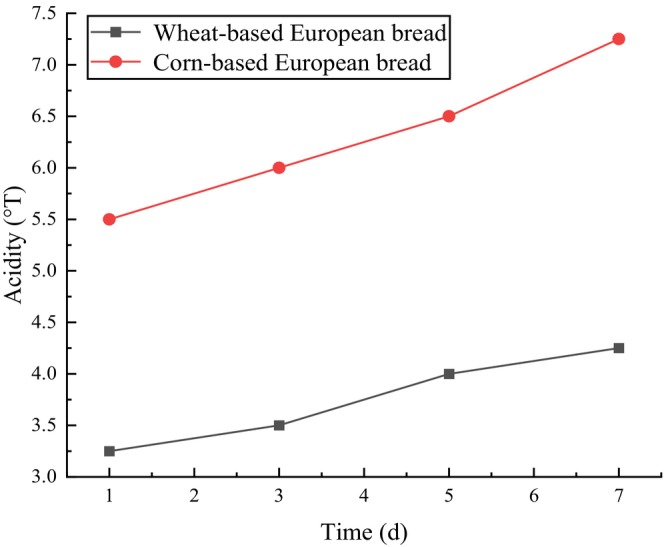
Acidity content of corn‐based and wheat‐based European breads during 7 days of storage. Each point represents the mean ± SD of three independent experiments (*n* = 3).

In addition, Figure 5 the acidity of both corn and wheat European‐style breads increased with storage time, with a more pronounced increase observed in corn European‐style bread. This behavior is closely associated with the incorporation of lactic acid bacteria during corn flour fermentation. Organic acids produced during lactic acid fermentation increased the initial acidity of the bread, while prolonged yeast growth and metabolic activity during fermentation further promoted acid formation. According to the Chinese national standard GB/T 5009.17‐2017, the acceptable acidity of bread should not exceed 6°T. Considering both acidity and textural changes, it can be concluded that corn European‐style bread maintains acceptable eating quality within 3 days of storage, whereas sensory and physicochemical quality deteriorate markedly beyond this period.

**FIGURE 6:**
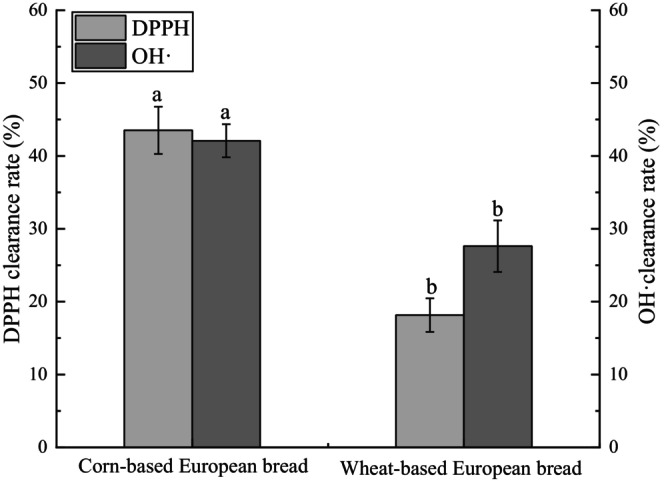
DPPH and hydroxyl radical scavenging activities of corn‐based and wheat‐based European breads. Letters in the bar graph indicate significant differences among samples (*p* < 0.05).

In Figure 6, compared with wheat European‐style bread, the corn European‐style bread extract exhibited significantly enhanced scavenging capacities against DPPH and hydroxyl radicals, increasing by 139.65% and 52.35%, respectively, indicating markedly superior antioxidant activity. This improvement can be primarily attributed to the abundance of natural antioxidant compounds in corn, including polyphenols, flavonoids, and carotenoids (Ibrahim et al. 2023). When whole‐grain corn flour is used as the main raw material, these functional components are largely retained during processing, allowing the corn European‐style bread to inherit and express the intrinsic antioxidant advantages of corn. Enhanced antioxidant capacity may contribute to the elimination of excess free radicals in vivo and thus holds potential nutritional value for mitigating oxidative damage and supporting chronic disease management.

In Figure 3, during in vitro simulated digestion, the starch hydrolysis rates of both corn and wheat European‐style breads increased with digestion time, exhibiting a rapid rise within 0–30 min followed by a slower increase thereafter, which is consistent with the general kinetics of starch digestion. However, compared with wheat bread, the starch hydrolysis rate of corn bread was significantly lower, with a reduction of 37.45% at 180 min. This difference is closely related to the compositional characteristics of corn bread: whole‐grain corn flour contains a relatively lower starch content with a more compact structure that is less susceptible to enzymatic digestion, while the higher dietary fiber content in corn bread forms a physical barrier around starch granules, thereby restricting enzyme accessibility and reducing starch hydrolysis efficiency (Huang et al. 2016).

Based on the area under the starch hydrolysis curve from 0 to 180 min, the glycemic index (GI) of the corn European‐style bread was calculated to be 67.45. According to the Chinese national standard GB 29922‐2013, foods with GI values between 55 and 75 are classified as medium‐GI foods. Accordingly, the corn European‐style bread developed in this study can be categorized as a medium‐GI food. Its consumption may help slow the rate of postprandial glucose release and prevent sharp fluctuations in blood glucose levels, thereby exerting a positive effect on long‐term glycemic control.

**FIGURE 7:**
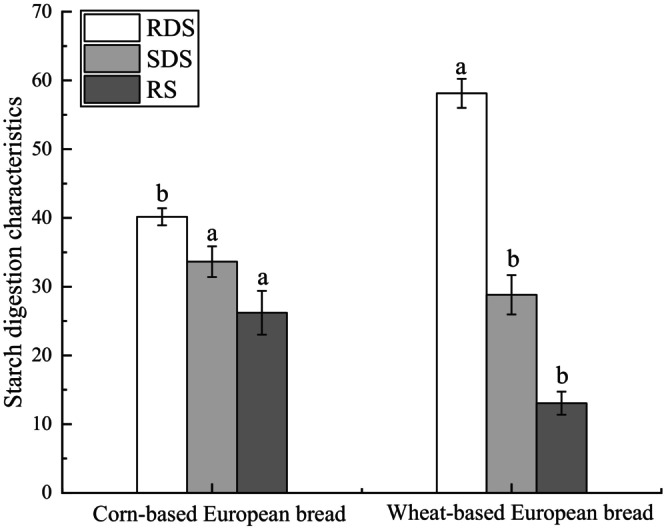
Comparison of starch digestibility between corn and wheat in European bread. Letters in the bar graph indicate significant differences among samples (*p* < 0.05).

In Figure 7, further analysis of starch fractions revealed that the rapidly digestible starch (RDS) content of corn European‐style bread was significantly lower, whereas the resistant starch (RS) content was markedly higher than that of wheat bread. This phenomenon can be attributed, on the one hand, to the higher dietary fiber content in corn bread, which encapsulates and protects starch within a fiber network, limiting enzymatic hydrolysis and increasing RS content to nearly twice that of wheat bread. On the other hand, the higher proportion of amylose in whole‐grain corn flour results in a more compact molecular arrangement, further enhancing starch structural stability and leading to a 118.55% increase in RS content compared with wheat bread (Du et al. 2022). Increased RS content not only reduces starch digestibility but also enhances satiety and exerts physiological functions in the gastrointestinal tract similar to those of dietary fiber.

## 5 Conclusion

This study demonstrated that the composite application of ultrafine grinding, extrusion puffing, and lactic acid bacteria fermentation effectively modified whole‐grain corn flour and improved the processing performance and quality attributes of corn‐based European‐style bread. The premixed modification strategy enhanced dough rheological behavior, loaf volume, antioxidant activity, and starch digestibility characteristics, while enabling the production of corn‐based bread with higher dietary fiber content and a medium estimated glycemic index.

However, this study was conducted at laboratory scale with a limited formulation range and did not account for biological variability among corn batches. Future work should focus on pilot‐ and industrial‐scale validation, as well as long‐term storage stability and sensory evaluation.

## Conflicts of Interest

The authors declare no conflicts of interest.

## Data Availability

The data that support the findings of this study are available on request from the corresponding author. The data are not publicly available due to privacy or ethical restrictions.
